# Intra-Abdominal Hydatid Cyst: Sociodemographics, Clinical Profiles, and Outcomes of Patients Operated on at a Tertiary Hospital in Addis Ababa, Ethiopia

**DOI:** 10.1155/2017/4837234

**Published:** 2017-12-12

**Authors:** Engida Abebe, Temesgen Kassa, Mahteme Bekele, Ayelign Tsehay

**Affiliations:** St. Paul's Hospital Millennium Medical College, Addis Ababa, Ethiopia

## Abstract

**Background:**

Hydatid cyst is caused by the tapeworm* Echinococcus granulosus*. The abdomen, specifically the liver, is the most common site affected.

**Objective:**

Determine the presentation patterns, types of surgical management, and outcomes of patients operated for intra-abdominal hydatid cyst (IAHC).

**Methodology:**

A retrospective descriptive study of patients admitted and operated for IAHC from September 1, 2011, to August 31, 2015.

**Results:**

Forty-two patients whose age ranged from 10 to 65 (mean of 37 years) were operated on. Females comprised 27 (64.3%) of the patients. The commonest presenting complaint was abdominal pain (41, 97.6%). Abdominal mass was documented in 23 (54.7%) cases. Abdominal ultrasound (AUS) and CT were the main imaging studies done on 38 (90.5%) and 24 (57.1%) patients, respectively. Cysts measuring more than 10 cm in diameter were the most common finding in both studies. Liver was the primary site involved, 30 (71.4%) cases, the right lobe being the main side, 73%. Thirty-eight (90.5%) patients underwent deroofing, evacuation, marsupialization, and omentoplasty (DEMO). There was no perioperative death, but 4 (9.5%) of the patients had post-op complications.

**Conclusion:**

Abdominal pain was the most common presenting complaint. AUS and CT remain the preferred imaging. DEMO was the most common surgery.

## 1. Introduction

Hydatid disease is caused by infestation by the tapeworm* Echinococcus* sp. Among the six known species of the tapeworm, two of them are frequent causes of hydatid disease in humans.* E. granulosus* causes hydatid cyst (HC), while* E. multilocularis* causes alveolar hydatid disease [1, 2]. HC is the most common form affecting humans. The disease is common in areas where humans, sheep, and dogs live in close contact. HC is endemic in Africa, Eastern Europe, Middle East, New Zealand, and Mediterranean region [1–3].

Humans are an accidental host for the tapeworm, while dogs are definitive hosts. Humans acquire the disease when they eat foods contaminated by the tapeworm [2]. HC affects both sexes equally, at all age groups, but is more often seen in middle-aged groups. HC can affect any part of the body, but the most commonly affected organs are the liver, the lung, and the brain [4–8]. Intra-abdominal hydatid cysts (IAHCs) in addition to the liver can be seen in the peritoneum, spleen, kidney, and pancreas [6, 7, 9].

Symptoms of IAHCs are dependent on the site, size, and stage of development of the cyst, whether the cyst is alive or dead, and whether complicated or not [10]. Because cysts grow slowly, in their early stage, IAHCs are asymptomatic. Common presenting symptoms for IAHC are abdominal pain usually localized to the right upper quadrant and abdominal masses [6, 10].

Diagnosis of IAHC can be ascertained by abdominal ultrasound (AUS) and/or CT scan [11, 12]. Classic findings on imaging of hydatid cysts are double layer thick cyst walls, often with daughter cysts [8]. Laboratory findings include eosinophilia, positive serology like enzyme-linked immunosorbent assay (ELISA), radioallergosorbent test (RAST), and the “arc 5” antibody test [11]. Aspiration of fluids by a fine needle (which is controversial due to the potential risk of anaphylaxis and spread of the disease) and histopathology are also helpful. Ultimate confirmation of the diagnosis is done by demonstration of parasitic elements in the surgical specimen [2]. The typical hydatid cyst has a three-layer wall surrounding a fluid cavity; the outer layer is called the pericyst, the mother layer is called the ectocyst, and the germinal layer is called the endocyst [2].

Treatment of IAHC can be medical, surgical, or in most symptomatic cases both. Percutaneous approaches are also options [13]. Options of surgical intervention depend on the size, number, and site of the cysts and include excision of the cyst, deroofing, cyst evacuation, obliteration, and marsupialization. At times, resection of part of or the entire affected organ may be necessary. Pre- and postoperative albendazole reduces the rate of recurrence [6, 14]. The aim of the current study was to determine the sociodemographic characteristics, presentation patterns, types of surgical management, and outcomes of patients operated on for intra-abdominal hydatid cyst at St. Paul's Hospital Millennium Medical College (SPHMMC).

## 2. Methods and Materials

A retrospective review of all patients operated on for IAHCs from September 1, 2011, to August 31, 2015, at SPHMMC was conducted from June to October 2016. SPHMMC is a referral tertiary level teaching hospital in Addis Ababa, Ethiopia. The hospital accepts referrals from all over the country. IAHC was defined as any HC found in the abdominal viscera, peritoneal cavity, omentum, retroperitoneal space, or organ. Operation theater log book and nurses admission and discharge book were used to identify patients. Individual patients' medical records were used as sources of data. Data including patients' sociodemographic characteristics, mode of admission, site of the disease, type of imaging studies done, type of operation performed, and outcome of patients were collected in a pretested data collection format by trained second-year surgical residents. The data was checked for completeness, cleaned, coded, entered, and analyzed with SPSS version 20. The associations of different variables when applicable were tested for significance in chi-square analysis. Considering a confidence level of 95%, a *P* value of <0.05 was considered significant in all statistical comparisons. Ethical clearance was obtained from SPHMMC Institutional Review Board.

## 3. Results

### 3.1. Sociodemographic Characteristics

A total of 44 patients were seen and the study included 42 (95.5%) patients with complete charts. The age of patients ranged from 10 to 65 with a mean of 37 years. The most commonly affected group was those in the fourth decade of life (Table 1). Females accounted for 27 (64.3%) of the patients, making the female-to-male ratio 1.8 : 1. Majority of the patients (62%) came from urban settings.

#### 3.1.1. Clinical Presentations

Most of the patients (38, 90.5%) were admitted on elective bases. Emergency admissions contributed only 4 (9.5%) of the cases. Abdominal pain was the main complaint at presentation (41, 97.6%), followed by nausea/vomiting and abdominal mass each reported by 19 (45.2%) patients. Abdominal pain was localized in the RUQ in 34 (81%) patients; the remaining 7 patients had either left lower quadrant, epigastric, or diffuse abdominal pain (Table 2). Physical examination showed that all of the patients (except patients with emergency admission) had vital signs within the normal limits. An abdominal mass was documented in 23 (54.7%) cases while 13 (31%) patients had hepatomegaly. In 16 (70%) of these patients, the mass was more than 10 cm in diameter, while 7 (30%) of the patients had a mass 5–10 cm in diameter. The common site for the abdominal mass was in the RUQ, accounting for 16 (70%) cases. Jaundice was found in 6 (23.3%) patients. Abdominal tenderness was seen and localized in the RUQ in 10 (23.8%) patients. Among the four patients admitted on emergency, three had tachycardia, fever, and rebound tenderness. One of the patients (female) presented with full-blown peritonitis with diffuse abdominal tenderness, guarding, and rigidity.

#### 3.1.2. Investigation Findings

Complete blood count was done for every patient and leukocytosis was found in 7 patients only. Liver enzymes were normal but 6 (14.3%) patients had elevated direct and total bilirubin. AUS and CT were the main imaging studies done, on 38 (90.5%) and 24 (57.1%) patients, respectively. Cysts measuring more than 10 cm in diameter were the most common finding in imaging studies, 60% in US and 71% in CT (Table 3). In both imaging studies, intracavitary daughter cysts were seen in 33.3% of the patients. Complicated cysts were found in 3 patients: one rupture to the general peritoneum and the others having communication with the biliary tree. Only one patient had abdominal MRI which showed a large multiseptated cystic mass in RUQ with rim enhancement. In another patient, MRCP revealed compression and elongation of right hepatic and common hepatic ducts and hypointense material filling common bile duct which was concluded to be HC. A diagnosis of HC was ascertained by the intraoperative findings of germinal layer and daughter cysts in 95% of the cases; in the rest, diagnosis was confirmed on histopathology examination.

### 3.2. Surgery

**Figure 1:**
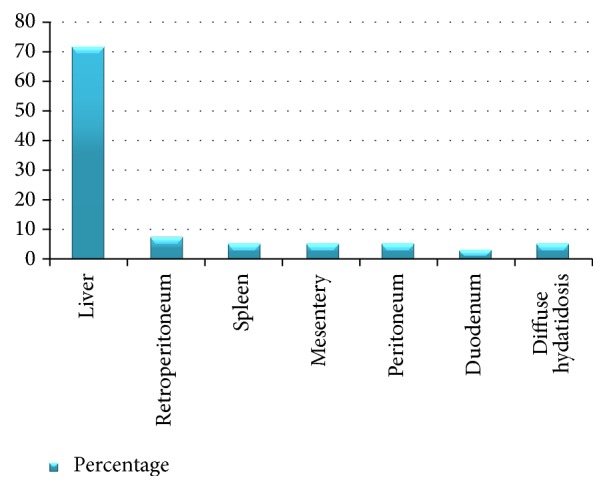
Intraoperative findings of cyst distribution by site.

Preoperative chemoprophylaxis (albendazole) was given to 33 (78.6%) patients for at least four days. The most common incision was right subcostal followed by a long midline incision. The liver was the primary site of the disease in 30 (71.4%) cases, the right lobe being the main side affected, 73%. Retroperitoneum was involved in 3 patients. Spleen, mesentery, and peritoneum were harboring the cyst as a primary site, each accounting for 2 (4.7%) of the cases as shown in Figure 1.

**Figure 2:**
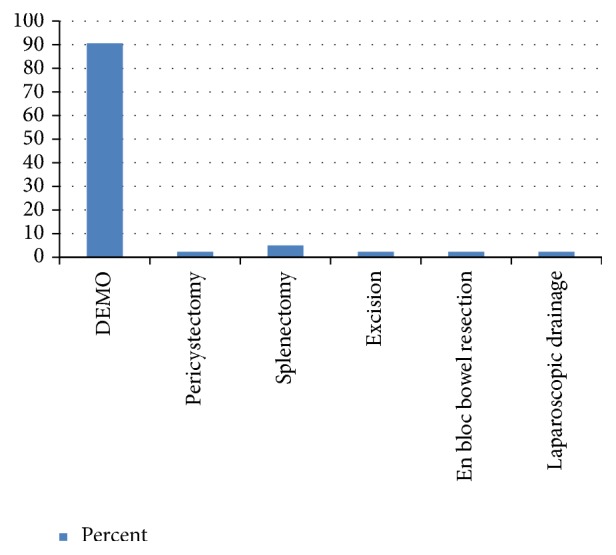
Types of surgery done.

The cyst had communication with the biliary tree in two patients and ruptured into the general peritoneum in one patient. Among the 42 patients, 4 patients had superinfected cysts with frank pus in the cavity. In all these cases, there were moderate to severe adhesions between the cyst and the surrounding structures. Thirty-eight (90.5%) patients underwent deroofing (of the cyst wall), evacuation (of the content), marsupialization (of the edge), and omentoplasty (obliteration of the cavity by placing omentum) (DEMO) Figure 2. None of the patients had perioperative anaphylaxis. Postoperatively, 37 (90.5%) patients took albendazole, and one patient was given mebendazole for an unspecified reason. Four (9.5%) of the patients had 6 postop complications: 4 surgical site infection and 2 bile leaks. Bile leaks stopped with follow-up with a drainage tube. All patients discharged were improved.

## 4. Discussion

This study showed IAHC to be a fairly common condition. Though the disease is considered to be common in rural settings, our finding showed a relatively higher proportion of patients to be from the urban setting [15]. Bilutse et al. from a different hospital in Addis Ababa also showed 60% of their patients with HC of the liver to be from urban settings [6]. This may be due to immigration of the patients from the rural to urban settings for economic reasons, which is very common in developing nations. The finding of young adults affected more commonly was also shown in literatures from Ethiopia and other developing nations [3, 6]. Studies from Bahir Dar, Northern Ethiopia, and Addis Ababa showed HC to be more common in the third decade of life (21–30 years) with a mean age of 37 and 33.5 years, respectively [3, 6]. Studies from Iran and Morocco found a mean age of 40 years and 39.5 years, respectively [10, 16].

Our finding showed a statistically insignificant female preponderance for IAHC like shown in Bahr Dar and Tehran studies where females made up 83% and 58% of the patients, respectively [3, 10]. Similar findings were also reported in studies from Saudi Arabia, Yemen, and Nepal [17–19]. Females acquire the disease more commonly than males probably due to their more intimate and frequent contact time with domestic animals (dogs and other pets) at home.

Like in the literature, abdominal pain, specifically right upper quadrant pain, was the main presenting complaint in our study [6, 10]. Bilutse et al. reported abdominal pain as presenting complaint in 84% of their patients with liver hydatid cyst [6]. Similarly, a study in Tehran showed among patients with hepatic HCs abdominal pain (60%) and RUQ pain (32%) to be the predominant presenting complaints [10]. This is easily understandable as one remembers that the liver is the most commonly affected organ by HC. Though the frequency of nausea/vomiting reported in these studies, 23.5%, was lower than our finding, it was still the second most common symptom of patients with IAHC.

Preoperatively, the diagnosis of hydatid cyst was made with imaging studies and confirmed by the intraoperative findings. The result of AUS and CT in this study was very good in defining the size, site, and nature of the cyst. The sensitivity and specificity of US in detecting HC reported in the literature were also very good [11, 12]. The rate of CT utilization was less than that of AUS. The reason for this could be that AUS was the first screening and diagnostic imaging which settled the diagnosis. Financial and availability issues can also be the reason for less utilization of CT. CT should be used only when AUS findings are inconclusive [12].

The finding of the liver in general and the right lobe of the liver in particular as the most common site involved in IAHC in our study is in agreement with the literature [3, 6, 10]. The study in Bahir Dar found 79.2% of the patients to have liver HC [3]. In the liver, the right lobe as the most common site affected was reported by studies from Addis Ababa, Tehran, and Morocco, which showed right lobe affected in 55.5%, 75%, and 50% of the patients, respectively [6, 10, 16]. Liver is the most commonly affected organ because it is the first organ which takes parasite infested portal venous system [2].

Though the majority of the patients had liver HC, the major physical finding was abdominal mass. Hepatomegaly accounted only for 30% of our cases. This is most likely due to the size of the cysts which were in excess of 10 cm in 60% of the cases which made defining the site of origin of the cyst difficult. This also shows how physical exam can be inaccurate in determining the site of origin of the cyst. Regarding cyst size, more than 95% of the patients had a cyst more than 5 cm in diameter. This is understandable as small cysts are asymptomatic and patients do not seek medical attention for that [2, 8, 10].

Nearly 30% our patients had extrahepatic intra-abdominal disease which was mainly seen in the retroperitoneum, the spleen, and the peritoneal cavity. Literatures show in the abdomen, next to the liver, the spleen and the kidney to be the two most common organs involved [3, 17]. The Bahir Dar study identified the spleen as the second most common site (20.8% of cases) of HC [3]. Literatures also reported HC in the adrenal glands, gall bladder, omentum, peritoneum, appendix, and ureter [8, 9, 19, 20].

Imaging and intraoperative findings identified 14.5% rate of complicated HC. The complication includes superinfection and ruptured cysts into the peritoneum and biliary trees. This figure is higher than the report in an Indian study where only a 5% complication rate was reported. The rate was higher in our study because all the patients were symptomatic at presentation unlike the Indian study which included asymptomatic patients. Like in the literature, rupture to the peritoneum and communication with the biliary tree were infrequent encounters [16, 21, 22].

The option of treatment for HC ranges from simple cyst drainage (evacuation) to resection of the whole or part of the involved organ [6, 23]. The major form of surgery performed in both liver and extrahepatic HC in our study was DEMO. Resection surgery was done only in few cases. This was mainly due to the nature of the cyst in which most are intraparenchymal liver cysts or peritoneal HC with extensive surrounding structure adhesions. Resection in such situation can result in unnecessary higher surgical complication rates. The outcome of patients treated with DEMO was also comparable to literatures [6, 23]. Though the preoperative scolicidal agent administration rate was low, the postoperative albendazole administration was good [24].

## 5. Conclusions

Abdominal pain and swelling are the two most common complaints of patients with IAHC. The liver was the most common organ involved, with the right lobe affected more often. AUS is a very good noninvasive and cheap imaging modality for patients suspected to have IAHC. Further imaging adds very little. DEMO is a relatively technically simple surgery which has excellent patient outcome.

## Figures and Tables

**Table 1:** Sociodemographic characteristics of patients operated on for intra-abdominal hydatid cyst, SPHMMC, 2015.

Features	Number	%
Age		
<20	5	11.9
21–30	8	19
31–40	11	26.2
41–50	9	21.4
51–60	5	11.9
>60	4	9.5

Sex		
Male	15	35.7
Female	27	64.3

Address		
Urban	26	62
Rural	16	38

Pattern of admission		
Elective	38	90.5
Emergency	4	9.5

**Table 2:** Clinical findings of patients operated on for IAHC, SPHMMC, 2015.

	Number	%
Complaints at presentation		
Abdominal pain	41	97.6
Nausea/vomiting	19	45.2
Abdominal mass	19	45.2
Appetite/weight loss	15	35.7
Fever	5	11.9
Hematuria	4	9.5
Jaundice	4	9.5

Physical examination		
Abdominal mass	23	54.7
Hepatomegaly	13	31
Abdominal tenderness	10	23.8
Jaundice	6	14.3
Chest finding	4	9.5
Fever	3	7.1
Splenomegaly	3	7.1
Ascitis	2	4.8
Guarding/rigidity	1	2.4

**Table 3:** Imaging results of patients operated on for intra-abdominal hydatid cyst, SPHMMC.

Characteristics	Description	Number	%
*(I) Ultrasound findings*			
Cyst size	>10 cm	25	59.5
	5–10 cm	10	35.7
	<5 cm	3	7.14
	Total	38	100

Cyst nature	Clear fluid, unilocular	7	18.4
	Clear fluid, multiseptated	8	21.0
	Total/partial separation membranes	2	5.3
	Intracavitary native cysts (daughter cyst)	14	36.8
	Solid-like mass	2	5.3
	Calcified walls	5	13.2
	Total	38	100

*(II) CT findings*			
Mass	>10 cm	**17**	**70.8**
	5–10 cm	5	20.8
	<5 cm	2	8.3
	Total	24	100

Cyst nature	Clear liquid image, unilocular	6	25
	Clear liquid, multiseptated	4	16.7
	Total/partial separation membranes	1	4.17
	Intracavitary native cysts (daughter cyst)	**8**	**33.3**
	Solid-like mass	0	0
	Calcified walls	5	20.8
	Total	24	100
